# Brain Recording, Mind-Reading, and Neurotechnology: Ethical Issues from Consumer Devices to Brain-Based Speech Decoding

**DOI:** 10.1007/s11948-020-00218-0

**Published:** 2020-04-30

**Authors:** Stephen Rainey, Stéphanie Martin, Andy Christen, Pierre Mégevand, Eric Fourneret

**Affiliations:** 1 Uehiro Centre for Practical Ethics, University of Oxford, Oxford, UK; 2 Department of Basic Neurosciences, Faculty of Medicine, University of Geneva, Geneva, Switzerland; 3 Braintech Lab (U 1205), Université Grenoble Alpes, Grenoble, France

**Keywords:** Neuroethics, Mind reading, Neuroprosthetics, Speech, Language, Philosophy, Neuroscience, Neurotechnology

## Abstract

Brain reading technologies are rapidly being developed in a number of neuroscience fields. These technologies can record, process, and decode neural signals. This has been described as ‘mind reading technology’ in some instances, especially in popular media. Should the public at large, be concerned about this kind of technology? Can it really read minds? Concerns about mind-reading might include the thought that, in having one’s mind open to view, the possibility for free deliberation, and for self-conception, are eroded where one isn’t at liberty to privately mull things over. Themes including privacy, cognitive liberty, and self-conception and expression appear to be areas of vital ethical concern. Overall, this article explores whether brain reading technologies are really mind reading technologies. If they are, ethical ways to deal with them must be developed. If they are not, researchers and technology developers need to find ways to describe them more accurately, in order to dispel unwarranted concerns and address appropriately those that are warranted.

## Introduction

This paper will explore ethical issues arising from neural technologies in terms of mind-reading. The term ‘mind-reading’ has been used to describe the mechanisms employed by brain–computer interfaces (BCIs), and neural decoding using neurotechnologies. In the philosophy of mind, the mind refers to mental states (imagination, emotions, intentions, perception, decision making, etc.), and with brain interfacing technologies, neuroscience is now able to highlight some correlations between mental states and cerebral activity. There is thus some material basis for the mind.

However, the access to some material basis of mental states remains piecemeal and does not embrace simultaneously all aspects of the mind. In other words, neural correlates remain physical imprints of the expression of the mind, but are not sufficient to be thought of as constituting the whole mind itself. Confusion should be avoided between mind and piecemeal thoughts, and between reading mind and reading some neural imprints of thoughts. In particular, neural prostheses may allow reading neural correlate fragments of mental states but not the whole mind on its global scale. The extent to which all the pieces of thoughts that can be decoded from neural recordings constitute whole thoughts thus remains unclear. First, we address this question generally, and then more specifically with reference to the context of a speech BCI. For the speech BCI we ask: to what extent might speech prostheses allow access to our thoughts?

While people in general are quite reliable in ‘reading the mind’ of one another, according to familiar behavioural and linguistic cues, they are actually mainly able to infer another’s thoughts based on the signs the other person externalises intentionally (subconscious and other such ‘tells’ notwithstanding). In particular, access to another’s inner thinking remains inaccessible, it being possible only to make predictions about that. A technological turn within this familiar practice excites ethical concern, since the type and content of information that one may access with a ‘mind-reading’ device may strongly diverge from human inferences based in more traditional interpersonal methods. Technology, perhaps, makes a tacit claim to be *objective* in a way that much interpersonal interpretation does not. Putting one’s mind in the realm of objective legibility may appear to include more jeopardy than putting it in familiar social, fallible, realms for this reason. The specific notion of technologically-mediated mind-reading is apparently a particular kind of concern, and so it requires a specific discussion.

In order to structure the enquiry as we go forward, we need to investigate:

1. What BCI and neural decoding can currently do, and what may be possible soon
2. Ethical issues in current and future neurotechnology
3. Speech neuroprosthesis as a mind-reading device

We will then ask, further:

4. How ought we to treat these ethical issues?
5. What further analysis is needed?

By getting a handle on the technological capabilities of neurotechnologies, like BCIs, we can realistically frame the ethical concerns that may arise. Likewise, in order to consider how ethicists ought to react to emerging issues, or pre-empt likely future issues, a clear picture of what happens in brain decoding contexts is necessary. Funding bodies, and the researchers they fund, have specific responsibilities here. In constructing calls for research, funders steer efforts in specific directions. In creating these technologies, researchers are relied upon to work responsibly, and to communicate clearly the nature of their work, both in terms of present capability and likely future scenarios. Ethicists need clear pictures of what is happening with funding strategies, and technology development, in order to reflect upon and respond to it. Ultimately, such reflections may go on to seed policy advice, as well as colour public perceptions. This is vital for a clear perspective on the research ecosystem, including how this impinges upon wider socio-political realities. We will explore this generally, as well as going deeper into a speech prosthesis as a special case. This is considered especially interesting given the proximity between much thought, and language.

## What BCI and Neural Decoding Can Currently Do, and What May be Possible Soon

At the most general level, neurotechnologies work by recording electrical activity in the brain, and applying various processes to the outputs obtained. Recording can happen within the brain itself via macroscopic or microscopic intracerebral/intracortical probes, on its surface with electrocorticography (ECoG), or from non-invasive electro- or magneto-cencephalography (EEG/MEG) recording devices positioned over the head. All types of brain recordings can be correlated with a variety of physical and cognitive activity.

Given the ongoing activity in the field of brain-reading and attempts to correlate this work with mental states, it is important to remain vigilant of social, legal and policy dimensions of primary research and concurrent technology development. The nature of the individual as an agent in their own right, a locus of intentional action, may be challenged in the development of technologies that appear to read minds, whether or not they actually read minds (Mecacci and Haselager 2019).

Reading the mind, like reading a book, implies something about mind’s being potentially open to view. This would mark a radical departure from conventional accounts of one’s mind as accessible only to oneself. In a mind-reading context, one person might gain access to another’s ideas, thoughts, intentional, emotional, perceptual states, or their memories. This might be done with or without permission. It could offer the promise of exciting new modes of communication, self-expression, and mutual understanding. Often the stuff of science fiction, this prospect can have alarming dimensions concerning who might have access to the mind, as well as implications for how persons might be judged. In a world of mind-reading ought a person to be judged in terms of what they reveal voluntarily, or what can be read from their thoughts?

For example, since 2013 it has been known that detection of a specific type of signal (the ‘P300’ wave) can play a role in ‘spying’ on brain activity to extract confidential information. This can be done with subliminal cues, perhaps to gain information predicting personal beliefs. Researchers constructed a game and recorded the brain activity of its players. These signals could be processed to elicit details about bank PIN numbers and related private information without the game player knowing (Ienca et al. 2018). This was done through recording brain activity during the game, and processing the signal to search for P300 waves in response to hidden cues. Brain data is thus clearly highly sensitive data because it can house information that a subject may not wish to externalise, but which may nevertheless become accessible by others, in specific situations using neurotechnology.

This point is raised quite acutely where neurotechnology would have applications in the legal sphere. Meegan (2008) discusses law enforcement applications of memory detection. Recognition of a scene, or an object, can be the sort of thing detectable in neural activity regardless of claims overtly made. As far as memory-reading goes, this might be seen as a litmus test—the idea of ‘guilty knowledge’ as a smoking gun in a courtroom. Would memories that are stored, but are not being reinstated at the present moment, be available to the mind-reader? This is a neuroscience question about how are memories stored, and the difference between a memory that has been stored and one that is being reinstated. It is also an ethical question, however, in that it has ramifications for what limits we ought to apply in treating them as readable in machine-like terms.

Through recording signals from various regions of the brain, research has suggested that quite fine-grained information can be partly read from brain activity. Motor plans, visual imagery, percepts such as faces (Chang and Tsao 2017), speech (Akbari et al. 2018) decision and intentions, landmark places, moods can all be predicted from neural recordings (Haynes et al. 2007; Kay et al. 2008; Roelfsema et al. 2018, p. 13; Sani et al. 2018). Existing research technologies can be used to decode the neural correlates of mental images too, the things seen by a person. In controlled circumstances, identification algorithms operating on fMRI data, can pick the image viewed by an experimental participant from a known set of exemplars. Experiments here can achieve over 90% accuracy (Kay et al. 2008). The idea of mental privacy certainly seems to be challenged by these kinds of activities. Such results appear to demonstrate that mental content can be ‘read off’ from brain measurements. This implies that though someone may be certain that they have unique, privileged access to their own thoughts, that certainty can be misplaced (Eickhoff and Langner 2019; Farah et al. 2009; see Mecacci and Haselager 2019).

If we want to focus on mind reading as a point of reference for ethical concerns surrounding neurotechnology, we can ask of the technologies and techniques mentioned here: Is this mind-reading? We would be compelled to answer, *not exactly*. In terms of the approaches to identifying mental images, for instance, the experimental protocol operates on the basis of a modelled receptive field, and activation data for sets of images. The images decoded from the fMRI data are selected from a known list, and represented as matching patterns of data. This is detailed and interesting work, illuminating much of how visual representations work in the visual system. But it isn’t the case that, in an uncontrolled environment, a device can reconstruct the visual experience of a given individual.

In the legal example, what can be said is that the techniques involve careful attention to specific neural activity in specific contexts. A memory can’t simply be ‘read’ as one could read a sentence on a page. This kind of memory detection exploits associations among known stimuli and evoked neural signals in order to warrant inferences about a subject’s past experiences or perceptions, like recognition of a particular image. If, when shown a crime scene, my brain exhibits a response associated with familiarity it may indicate that I was there.

Clearly, there are risks and potential for false positives with this kind of approach. On the other hand, it seems equally clear is that the idea of accessing the real content of memory, or downloading a set of memories, doesn’t come up. But this does not mean that ethical problems do not arise, however. Where some practice might be *taken as* mind reading, we ought not to be too complacent in having ruled out ‘real’ mind reading on a technicality. An approach sensitive to ethical, and socio-political realities is required in order to deal with the possibilities for pseudo-mind reading in which people may fall prey to bad practices.

## Ethical Issues in Current and Future Neurotechnology

To the extent that neurotechnologies embody somehow a claim that the mind may be open to view, they each raise ethics concerns relating to a range of issues, including mental privacy. Relating to this too, is a concern over the reduction of mental states to sets of neural data. We will get into more detail on these and the related areas of cognitive liberty and self-conception. Before delving into these functional issues arising from the use of neurotechnology, something should be said about the presentation of neurotechnology.

Outside the research lab, there is a variety of BCIs already available commercially, including products like Cyberlink, Neural Impulse Actuator, Enobio, EPOC, Mindset (Gnanayutham and Good 2011). The potential prospects for applications based on these types of technology are interesting (Mégevand 2014). However, the plausibility of technological claims ought to be carefully scrutinised.

While the detection of neural signals is in principle easy, identifying them is difficult (Bashashati et al. 2007). A lot of research effort aims at improving detection and recording technology. This should help to improve the prospects for identifying recorded neural signals. Identification is centrally relevant to mind reading in that the signals recorded must be correlated somehow with mental states. It is ethically relevant too, not least owing to the prospects of misidentifying mental states via inappropriately processed brain recordings, or through misrepresenting the nature of the recording taking place.

Brain signals can be sorted into types. Recording sites can be classified in functional ways—visual, motor, memory, language areas, for example. That types of signals in specific areas appear to be ‘behind’ our conscious activity suggests that activity ought to be classifiable in a quite objective way. At least some neurotechnological development paradigms would suggest that this was the case: claims have been made about the kinds of technologies discussed above as ‘accessing thoughts’, ‘identifying images from brain signals’, ‘reading hidden intentions’ (Haynes et al. 2007; Kay et al. 2008). Attending to the brain signals means getting to the mental content, these claims suggest.

But this may be a case of overclaiming. It seems as if a great deal more information than is captured through measuring brain signals is required if meaningful inferences about thought content are to be drawn from them. For example, Yukiyasu Kamitani carried out experimental work aimed at ‘decoding dreams’ from functional magnetic resonance imaging (fMRI) data. Media reports presented this work as if dreams were simply recorded from sleeping experimental participants (Akst 2013; Revell 2018). But in reality, 30–45 h of interview per participant was required in order to classify a small number of objects dreamt of. This is impressive neuroscience experimentation, but it isn’t it just a ‘reading of the brain’ to ‘decode a dream’. Interview is an interesting supplement to brain signal recording because it specifically deals in verbal disclosures about the experience of mental states.

When it is reported that Facebook or Microsoft will develop a device to allow users to operate computers with their minds or their thoughts (Forrest 2017; Solon 2017; Sulleyman 2018), this is perhaps a too-extravagant claim. While many consumer devices are marketed as ‘neurotechnology,’ it is implausible that they actually operate via detecting and recording brain signals (Wexler and Thibault 2018). Far more likely is that such devices will respond to electrical activity in the muscles of the face, the signals in which are maybe 200 times as strong as those in the brain, and much more closely positioned to the device’s electrodes. In all likelihood, doing something like typing with such a device exploits micro-movements made when thinking carefully about words and phrases. Muscles used in speaking those words are activated as if preparing to speak them, hence corresponding to them in a way that can be operationalised into a typing application. Indeed, this is the stated mode of operation for Google’s ‘AlterEgo’ device (Kapur et al. 2018; Whyte 2018).

Overclaiming is an ethical issue as it can undermine confidence in neurotechnologies in at least two ways: failing to deliver by misrepresenting technologies, and serving to raise undue hopes and concerns. This builds on a misleading representation of how a device works, and its prospects as an effective technology. There are ethical implications from this in terms of user consent in using a device. There may be varying degrees of deception at work, given this sort of misrepresentation, that could affect how we ought to consider the potential uptake and use of devices, whether by experimental participants or consumers.

Drawing on the dream decoding example, we have reason to think that the objective recording of brain signals is insufficient as an account of a mental state precisely in that it has no experiential dimension. Thoughts occur within an internal model of the world from a particular point of view. This model cannot be straightforwardly generalised from subject to subject based on brain signal observation. Only specific dimensions of this model can be inferred, limited in terms of predictability, and only after large amounts of training in contexts of rigorous research conditions. The objective promise of recording brain signals might be exactly what cuts them off from the mind, which includes a subjective perspective.

The possibility of a too-zealous reduction of the mind to some neural data arises here as an ethical concern. ‘Mental’ concepts can bear discussion without reference to ‘neuroscientific’ concepts (also vice versa). How each might relate to natural kinds is an open question (Churchland 1989). There is therefore a ubiquitous question of interpretation to be remembered, as the interplay between mind and brain is considered. The thought-experiment of a ‘cerebroscope’ serves to highlight this.

The cerebroscope is a notional device that records all activity of all neurons in the brain on a millisecond by millisecond basis. With this total representation of neural activity, the question is whether we have a representation of the mind. Steven Rose suggests not—the nature of the brain as an evolving, plastic entity, means that millisecond by millisecond resolution of neural activity is not intelligible without a total map of the genesis of those active neurons and their connections:

…for the cerebroscope to be able to interpret a particular pattern of neural activity as representing my experience of seeing [a] red bus, it needs more than to be able to record the activity of all those neurons at this present moment, over the few seconds of recognition and action. It needs to have been coupled up to my brain and body from conception—or at least from birth, so as to be able to record my entire neural and hormonal life history. Then, and only then, might it be possible for it to decode the neural information. (Choudhury and Slaby 2016, p. 62ff)

We should be careful in considering these sorts of issues when it comes to thinking of mind-reading. It might be thought that the mind is akin to a space through which a putative mind-reader could walk, and examine what is to be found in there. But Steven Rose’s point suggests a more situated kind of mind, reliant upon its genesis as well as its state at some moment in time. The point being made is that even were one to perceive the thought of another somehow it could only be understood as a subjective thought, not as an objective thought had by another.

Relatedly, Mecacci and Haselager (2019) discuss some philosophical ideas that relate to the privacy of ‘the mental’. They describe a perspectivalism from A. J. Ayer regarding mental states, prioritising the privacy of the mind and its contents. Such a view would also appear to rule out mind-reading as they require a particular perspective, meaning they appear not as objects in a mental space potentially open to view, but private contents of a specific mind.

Misrepresentation of technology, and reductionism, each appear to be dimensions of ethical importance in themselves. But a little more analysis of each shows them to lead to a broader set of ethical issues in neurotechnology. Where mental privacy is threatened, cognitive liberty may suffer. ‘Cognitive liberty’ includes the idea that one ought to be free from brain manipulation in order to think one’s own thoughts (Sententia 2006). This concept often arises in the context of neuro-interventions in terms of law or psychiatry, or neuroenhancement (Boire 2001). Here, it is most salient in connection with a potential loss of mental privacy.

Where mental privacy is uncertain, it is not clear that someone may feel free to think their own thoughts. Where measurements of brain activity may be taken (rightly or wrongly) to reveal mental contents, neurophysiology itself could be seen as a potential informant on thought itself. This would be to uproot very widely assumed notions about a person’s unique and privileged access to their own thought. If a keen diarist was to become aware that their diary could be read by another, they might begin to write less candid or revealing entries. If anyone became sure that measurements of their brain might reveal any of their mental contents, how might they refrain from having candid and revealing thoughts? This would amount to a deformation of normal ways of thinking, in rather a distressing way.

With this distressing possibility, the very idea of self-conception too is threatened. Where mental privacy concerns lead to inhibition of cognitive liberty, it would not be certain that one might feel free to reflect upon values, decisions, or propositions without threat of consequences. Considering ethically dubious thoughts, even if one considered them only to develop ways to refute them, might become dangerous where the content of the thought might be read from the activity of the brain. Faced with technology that appears to read minds, it seems ethical risks are posed by that technology in representing the mind as open to view.

Part of what it is to have a mind, and to be an agent at all, able to act on one’s reasoned opinions, includes reflection. This might mean that we wish to consider things we wouldn’t do, run through options we may disdain, or otherwise wish to reject. If we were to find ourselves in a context where mental contents were thought of as public, this reflective practice could suffer. Especially where such mental data might be held to be a more genuine, unvarnished, account than one offered in spoken testimony. This might build upon the principle at stake in the ‘guilty knowledge’ example from above. A chilling effect on thinking itself could materialise owing to the possibility of very intimate surveillance via brain recording.

The mediation of thoughts, ideas, deliberations, into actions is part of autonomous agency and self-representation. The potential for indirectly representing such things in one’s action is part of what makes those actions one’s own. Where a mind-reading device could be imagined as ‘cutting through’ the mediation to gain direct access to mental contents, this would not necessarily make for a more accurate representation of a person. Nor might it underwrite a better explanation of their actions than an explanation they might volunteer. At the heart of this is the privacy of mental activity, and the space this allows us to deliberate. Nita Farahany has called this a ‘right to cognitive liberty’ (Farahany 2018).

The privacy of deliberation is very important in providing room for autonomy, and substance for agency. As has been mentioned, inner mental life can be characterised to a greater or lesser extent through one’s behavioural cues. The difference between the reluctant carrying out of a task, as opposed to an enthusiastic embracing of the same is often fairly obvious. But indirect assessments of someone’s state of mind in their activities is a familiar, fallible, and well-established interpersonal practice. The idea that objective data might be used to directly characterize an attitude, once and for all, serves to undermine the role of agency. A decision to act represents a moderation of impulses, reasons, desires. If a mind-reading device were deployed it would represent a claim on the *real* state of a person’s mind, certainly. But this could serve to downplay that person’s action as more complex than simply the outcome of a neural process.

Thinking of the cerebroscope example, this is akin to the decontextualisation of neural recordings discussed there. The nature of the signals represented may make little sense outside of a biographical story. They may be likely, thereby, to misrepresent the person recorded. The fact that extensive testimony played such a large part in the dream reading experiment seems to back up this thought-experimental conclusion.

More broadly, it is important to discuss the purpose to which mind-reading devices are put. For instance, wearing a cast on a broken arm displays some dimensions of a person’s physiological state. However, this is of low concern because no one might gain from wanting to ‘steal’ such information. But what is problematic is the potential for the misuse of people’s thoughts, choices, or preferences as inferred from neurotechnology. Even if thoughts are not accessible by a technology, but it is possible that they are taken to be so, ethical issues arise. With a commercialisation of neurotechnology as ‘mind reading’ technology, these potentialities multiply as technology may be deployed where there is no particular need. This leaves open a question about what purposes the technology may be used for, and by whom. A potential diversity of technologies, actors, purposes, and stakes make for a complex picture.

The socio-political ramifications of widespread neural recording could be deep. From these recordings, detailed predictions can be made about private, intimate aspects of a person. For those with access to it, this data will be a valuable asset. Facebook’s intended brain–computer interface, permitting seamless user interfaces with their systems would not only record and process brain signals, but associate the data derived from them with detailed social media activity (Robertson 2019). This would represent a valuable resource, providing rich links between overt actions and hitherto hidden brain activity. This kind of detailed *neuroprofiling* will likely be taken to be as unvarnished and intimate an insight into a person as it is possible to acquire. To the extent that this is accurate, new dimensions of understanding people through their brains might be opened. As with the political micro-targeting scandals involving Facebook and Cambridge Analytica, this data can also enable personal manipulation, as well as social and political damage (Cadwalladr and Graham-Harrison 2018).

At the personal level, databases that associate not only behavioural, but also brain data, represent serious risks for privacy and wider dimensions relating to dignity. The kinds of profiling they would enable would risk marginalising individuals and groups, while eroding solidarities among diverse groups. This happened in the run up to Brexit, based in covert psychometric profiling, and has had lasting social damage (Collins et al. 2019; Del Vicario et al. 2017; Howard and Kollanyi 2016). Targeting information at specific individuals or groups based the neural data would represent a new front in data-driven marketing or political campaigning, enabling novel, more sinister, and perhaps harder to deflect, forms of manipulation (Ienca et al. 2018; Kellmeyer 2018).

These examples focus upon how information can be leveraged for specific effects. Where neuroprofiling converges with advancing technology, direct neural-based manipulation also arises as a potential concern. Among the types of neurotechnology already available for research and for consumer purposes are those that use brain data to control software and hardware, those that display data for user’s purposes as neurofeedback, and those that seek to modify brain activity itself. These neurostimulation or neuromodulation devices use data derived from the brain to modulate subsequent brain activity, regulating it according to some desired state (Steinert and Friedrich 2019). This is quite a clear challenge to autonomy. Outside of an ethically regulated context such as that of a university research lab, this ought not to be taken lightly. Market forces are not self-evidently sufficient for ensuring the responsible marketing, and use, of such potentially powerful devices.

The kinds of concerns being discussed here are not based in mind-reading per se, but rather in effects likely to occur in the context of widespread neurotechnology use. Beyond the market context however, in the realm of ongoing research, at least one sort of mind-reading might appear to be technically possible in a limited sense at least. Following analysis of this case, we will be well placed to take a position on the ethical concerns that have arisen across a variety of applications from those where mind-reading is not the central effect to one in which it would be most likely.

## Speech Neuroprosthesis as a Mind-Reading Device

A first impression might be that ‘thought’, to the extent that thought can be ‘in words’, is substantially linguistic. While all thought is not necessarily something verbal: images, sounds, smells, etc. can be brought to mind as well, significant dimensions of thought such as internal monologue, or inner speech are readily conceivable as *thinking in words* (Perrone-Bertolotti et al. 2014).

This does not sound so far away from some of the explanation of human consciousness provided by Dennett (1993). On his account, augmentations upon abilities and instincts evident in many animal species are at least partly realised in human beings through linguistically borne ‘microhabits of thought’. For Dennett, this is what turns a *brain* into a *mind*. If language plays these kinds of roles, perhaps even being constitutive of minds as we know them as Dennett appears to suggest, ‘inner’, ‘silent’, or ‘covert’ speech may be very close to mental contents. What’s more, these kinds of non-externalised speech signals can be recorded from the brain. In the recording of covert speech, there is some *prima facie* possibility of technology-mediated thought-reading.

Whereas in natural speech, the vocal cords create a vibration that is modified by the vocal tract to create a word (or phoneme, or syllable), a neural-based speech processor takes as input neural signals, applies a modifying function, and creates a new signal as output. Such systems record the neural signals associated with vividly imagined, but unverbalised speech, and translates these signals into intelligible speech without any need of peripheral nerves or muscles activation. Currently, several strategies have been investigated to define what is the best speech representation to be decoded to target this type of speech interface.

One strategy is to classify the neural activity into a finite number of choices. Several studies have shown the feasibility to decode discrete units of speech, such as phonemes (Brumberg et al. 2011; Ikeda et al. 2014; Pei et al. 2011) or words (Martin et al. 2016), during covert speech.

If every mental state correlates with, or is realised by, a neural mechanism, then reading signals from the brain ought to allow access to mental states, including covert speech states. Covert speech seems a contentful medium, and one that really could be decoded in a mind-reading scenario. In terms of research-grade neurotechnology, in the context of controlled conditions, devices that are triggered by covert speech activity could be triggered by mentalised speech not intended for externalisation (Bocquelet et al. 2016). Considering further decoding techniques, especially the use of artificial neural nets, this could further be compounded as neural activity associated with types of covert speech activity might be discerned in a way that bypasses the user’s intentions.

Building software that directly maps the neural activity to any speech representation remains difficult due to the lack of any measurable behavioural output during covert speech, however. An alternative solution is based on the fact that imagined, covert speech, has features like those associated with the neural correlates of overt speech (Bocquelet et al. 2016; Chakrabarti et al. 2015). As such, it becomes possible to build a decoding model from an overt speech condition, and then apply this decoder in the covert speech condition to reconstruct acoustic speech features (Martin et al. 2014). Studies demonstrate the feasibility to decode basic speech features from neural signals during covert speech, but also emphasize the difficulty in extracting the patterns accurately. This illustrates how far we currently are from developing a sci-fi mind-reading device.

In principle, more brain signals than intended could be recorded in the kind of system just outlined. From any recorded signal features of relevance must be extracted such that they create an appropriate source for the modifying function. Means of determining speech-relevant source signal features might include the use of machine learning, using probability functions for each phoneme in a given language (Amodei et al. 2016; Hinton et al. 2012). This kind of approach would recognise language-relevant neural signals in terms of a mapping between neural signal and likely phonetic correlates.

Recalling the relations between thought and speech it seems possible that a too-sensitive speech device, based in covert speech, could externalise some parts of a person’s *internal monologue.* In some sense at least, this could be a case of mind-reading, perhaps not as generally represented in sci-fi, but nonetheless an example of internal monologue being externalised by technical means. One of the main conceptual, technological, and ethical difficulties here is to distinguish the covert speech that should be externalized from that which should not.

What’s more, with the inclusion of machine learning, language models could be integrated such that phonemes in a language could be predicted based on the model. This would mean that, as well as brain signals, a language model also adds a predictive dimension to the speech prosthesis system. In principle, the system could ‘guess’ the words to be spoken before the biosignals are realised that would coincide with the phonetic signal. The prediction could be done well but, in being based on neural signals and model-based predictions, nevertheless occur in the absence of a decision to speak out loud. This could be as if the system were speaking on the user’s behalf, perhaps undertaking delegated action without express permission (Rainey 2018).

In any case of speech prediction, there could be the problem that the system could externalise something not intended at all by the user, not as thought or as speech. Even where a robust system of retraction was in place, there would be a risk that erroneous speech was *taken as* that of the user. This could amount to a challenge to their first-person authority.

So, in terms of imagined speech, there is an obvious risk in principle. The nature of the recording and decoding, in being triggered by covert speech, could feasibly result in more speech being externalised than expected or desired. This could be because of the way triggering works as based in brain signals and predictions from language models, prior to conscious decisions to act (Glannon 2016, p. 11). This raises some prospect of thought-reading, based covert speech involuntarily captured by a brain signal recording.

## How Should These Ethical Issues be Treated?

User control over neurotechnologies would appear to be of great importance in mitigating the potential mind-reading risks to privacy, autonomy, agency, and self-representation. A fine-grained ability for the user to select what exactly is output by such devices would be a good start. Besides this, some ability to retract actions mediated via brain controlled devices ought to be built in. This ‘veto control’ (Steinert et al. 2018) would allow for some practical distinction to be made between brain recording-related disclosures to be considered deliberate or not. This might be most obviously illustrated with reference to a speech device. In term of a speech neuroprosthesis, speech action and the output of involuntary or other proto-speech act elements (e.g. thinking things through verbally), ought to be strictly user-controllable. Speech that the user intends to broadcast verbally should be clearly distinguishable from inner speech that the user does not want to broadcast. The user ought to have strict control over this distinction.

More than this, however, regulatory systems must be put in place to anticipate neurotechnology-specific issues. These will include not only how neurotechnologies are presented, but also how they work, and what sorts of applications they ought to be circumscribed from. For instance, medical device regulation, and data protection regulation, are likely each deficient when it comes to consumer neurotechnologies (Allison et al. 2007; McStay and Urquhart 2019). Devices of that sort are not medical, yet they might operate on health-relative neural functions, and record and transmit health-relative data. The developers of brain technologies ought to, as part of their product or application development, maintain active links with policymakers in order that appropriate regulation can be framed.

To illustrate, it is likely that private companies will drive much neurotechnology development, even in assistive applications. To some extent, some users will thereby be relying upon those private companies in order to be able to live a fuller life, whereas others will use devices more recreationally. How assessments may be made of this kind of distinction in action, between those who cannot act but for a device and those who merely choose so to act, represents a novel issue. Policymaking will be required for scene-setting around the introduction of devices that introduce this distinction, highlighted by ethical analysis. This would be a useful, and ethically sensitive, means of anticipating near-future issues in conjunction with technology development.

## What Further Analysis is Needed?

The technology to routinely, accurately, record all of the brain signals required to reconstruct something like a stream of consciousness is not yet here. Nevertheless, neurotechnology is a burgeoning field, and techniques, materials, technologies, and theories are being refined at a pace. Anticipation of future developments ought to become live research ethics focal points in neuroscience and related labs in order to avoid a ‘delay fallacy’, as discussed in Mecacci and Haselager (2019).

Given the sorts of high stakes possibilities described here, we might do well in developing neurotechnologies to consider the benefits proposed applications will deliver. If we can answer the question *why do we want this neurotechnology now*, we may have good reason to proceed. If we cannot, be may have good reason to pause. ‘We’ here will include a variety of actors, it should be noted. Asking and answering the question *why* will likely be a very widespread discussion, drawing upon a variety of expertise, social, political, legal, and ethical resources. That such a discourse is so complex ought in itself to indicate the pressing nature of questions surrounding neurotechnological advance.

Specifically in terms of the thought-reading speech neuroprosthetic case discussed here, and other such assistive neurotechnologies, the question *why* is most clearly answerable. Where disability or disadvantage can be alleviated well with technology there is a strong case to be made for its development. Ethical issues that do arise cluster around the concept of control, in order to protect the volition of technology users. These concerns can be mitigated by sensitivity constraints within the system, and veto control whereby a user can halt entirely the synthetic speech emanating from their speech device. Conceptual analysis of the nature of responsibility ought to be used to inform technological development in terms of device activation, control, and veto, in order to ensure voluntariness remains central in device use. These relate to device-centred concerns that may emerge. On a wider perspective, how outputs from devices are received by audiences, are dealt with in law and policy, and feature in social perspectives, requires some thought.

In relation to user control over neurotechnologies in general, developers should ensure that any BCI affords the user as much control as possible, with a focus on reliably distinguishing between intentional triggering and neural activity merely sufficiently like it to cause device activation. These kinds of ethical dimensions even the more clear-cut case of neurotechnology for virtuous purposes, illustrate likely areas where subsequent problem could arise. Legal ramifications of devices not sufficiently and demonstrably in the control of users are likely to arise where ethical issues surrounding responsibility for technology-mediated action are not treated as the technology develops. Where a user relies upon their device, moreover, it will be vital that this somehow be taken into account in terms of the functioning of a device.

A further area likely to require more ethical, and legal, analysis will be that of data. Neurotechnologies will operate on the basis of a lot of brain derived data. This is sensitive material, from which can be inferred a range of health and other personal information. Yet the relations between data and persons requires further clarity (Rainey et al. 2019). In some senses, we are our data, but to a substantial degree we are not, being merely represented by it in particular ways, relative to the purposes for which it was collected, the means used toward that collection, the mode of storage, and so on. But how this works is a matter in need of debate, as illustrated in issues surrounding the use of Big Data (Bollier and Firestone 2010; Boyd and Crawford 2012).

At any rate, we ought not to proceed with neurotechnology developments that will raise data questions, and then try to work it out. Too much potential risk of different kinds would attend that approach. Especially where databases including brain derived data are already being created, the very existence of such resources is a problem where no clear conceptualisation is ready for their nature. Data privacy is emerging as a collective concern (Véliz 2019). As the science advances, it is through interdisciplinary discourse, highly reflexive and inclusive discussions that policy, legal, and social norms can be kept up to date. These technologies represent challenges to which we ought to respond in constructive ways in order that research and citizens alike be safeguarded.
